# Real-World Data from the First Intracranial Aneurysm Cohort in the Eastern Caribbean from 2021 to 2024: The Population Characteristics, Treatment Outcomes, and Effectiveness of the Newly Established Regional Organization with Air Transfer to the First Tertiary Neurointerventional Center in the Eastern Caribbean

**DOI:** 10.3390/jcm14134565

**Published:** 2025-06-27

**Authors:** Thibaud Pesce, Aboubacar Keita, Thomas Agasse-Lafont, Marie Sabia, Francois Barbotin-Larrieu, Dabor Resiere, Stephanie Puget, Moustapha Drame, Christina Iosif

**Affiliations:** 1 Department of Diagnostic and Interventional Radiology, University Hospital of Martinique, 97261 Fort-de-France, French Antilles, France; thib95.p@gmail.com (T.P.); aboubacar.keita@chu-martinique.fr (A.K.); thomasagasselafont@gmail.com (T.A.-L.); 2 Intensive Care Unit, University Hospital of Martinique, 97261 Fort-de-France, French Antilles, France; marie.sabia@chu-martinique.fr (M.S.); dabor.resiere@chu-martinique.fr (D.R.); 3 Department of Anesthesiology, University Hospital of Martinique, 97261 Fort-de-France, French Antilles, France; francois.barbotin-larrieu@chu-martinique.fr; 4 Faculty of Medicine, Campus Site de Martinique, University of the Antilles, 97157 Pointe-à-Pitre, French Antilles, France; stephanie.puget@chu-martinique.fr (S.P.); moustapha.drame@chu-martinique.fr (M.D.); 5 Department of Neurosurgery, University Hospital of Martinique, 97261 Fort-de-France, French Antilles, France; 6 Department of Clinical Research and Innovation, University Hospital of Martinique, 97261 Fort-de-France, French Antilles, France

**Keywords:** intracranial aneurysm, subarachnoid hemorrhage, coiling, stent-assisted coiling, endovascular treatments

## Abstract

**Background/Objectives:** The establishment of the first tertiary Neurointerventional Center at the University Hospital of Martinique in 2021, with full coverage of the populations of the French Antilles and Guyana, represents a paradigm shift in the treatment of intracranial aneurysms in the eastern Caribbean. We sought to evaluate the outcomes of the first cohort of patients treated for intracranial aneurysms from 2021 to 2024. **Methods:** We analyzed demographic, clinical, and angiographic data from a prospectively maintained database of patients treated from 1 January 2021 to 31 March 2024. The primary endpoint was the clinical outcome (mRS at discharge and at 4–6 months), and the secondary endpoint was the angiographic outcomes. **Results:** One hundred patients (mean age 56.7 ± 12.2 years old) with a total of 125 aneurysms (60.8% ruptured; 39.2% unruptured) were included from the following regions: 60% from Martinique, 21% from Guadeloupe, 13% from French Guyana, 1% from mainland France, 2% from St Martin, and 3% from abroad. The mean initial GCS value was 11.6 (median: 13; min: 4; max: 15); the mean mRS was 1.8 ± 1.7 before intervention, 1.8 ± 2 at discharge, and 1.7 ± 2 at 4–6 months. A total of 75% of the aneurysms were treated with coiling or remodeling, 23% received stents (20% FDs), and 0.8% were treated surgically. The procedure-related morbidity rate was 5.6% (7/125), and the mortality rate was 10.4%; both these percentages concerned only the ruptured cases. In the ruptured aneurysm subgroup, 32.8% (25/76) of complications were SAH-related, 9.2% (7/76) were hydrocephalus incidences, and 23.6% (18/76) were vasospasm cases. Satisfactory occlusion was obtained for 95.2% of the aneurysms post-procedure and for 96.7% at the last angiographic control. At the six-month control, 68% of the patients were independent in their everyday lives (mRS ≤ 2). **Conclusions:** The population was distinct in terms of the hyperexpression of risk factors, the multiplicity of IAs, and the severity of SAH. Female predominance was higher than usual in the population (81%). The organizational schema seemed effective; the treatments were safe and effective in terms of the clinical and angiographic outcomes.

## 1. Introduction

Intracranial aneurysms (IAs) represent a substantial risk for patients due to the high morbidity and mortality rates when ruptured. Both emergency endovascular management as well as elective treatments are mandatory in order to alleviate the burden of handicap and death due to this pathologic entity, which has a female predominance. In the French Caribbean territories, there is an overexpression of factors predisposing people to neurovascular pathologies, especially hypertension, a known factor for the development and rupture of IAs [1,2,3,4].

At the same time, there is a documented lack of interventional neuroradiology services in the Caribbean islands, revealed by the WHO and other scientific societies, which vary greatly among nations [5]. In the French Antilles, composed of the French islands of Martinique, Guadeloupe, Saint Martin, Saint Barthelemy, and their neighboring islets, as well as French Guyana, patients with acute intracranial aneurysm rupture were managed by transfer to mainland France, mainly Paris, for treatment.

This paradigm changed in 2021 initially by the rotation of interventional neuroradiologists (INRists) from mainland France, and then later in the same year with the onsite installation of an experienced, academic interventional neuroradiologist at the University Hospital of Martinique and the training of medical and paramedical teams in order to offer a full range of INR services [6,7].

We sought to evaluate this paradigm shift in terms of the safety and effectiveness of the management for patients from the French Antilles–Guyana regions in order to adjust the future strategic directions of territorial organization, including personnel and infrastructure resources.

## 2. Materials and Methods

### 2.1. Territorial Organization

The French West Indies region, with a population of slightly over one million inhabitants, is composed of four main islands: the islands of Guadeloupe and Martinique with 388,727 and 365,734 inhabitants, respectively; Saint Martin (32,358 inhabitants); and Saint Barth’s (10,585 inhabitants), as well as the continental region of French Guyana (294,071 inhabitants). The first complete tertiary INR department in the area was established in 2021 at the University Hospital of Martinique, which serves the other areas by air transfer.

Patients living outside of Martinique are transferred by air in cases of SAH (via HEMS, the helicopter emergency medical service from Guadeloupe and the neighboring islands, and commissioned commercial flights from Guyana) within 24 h for patients from Guadeloupe and the neighboring islands and within 72 h for those from French Guyana. They spend the necessary amount of time in the intensive care unit of the hospital in Martinique, with a minimum of 2 to 3 weeks for ruptured aneurysms.

SAH-related complications are managed onsite for all patients, including ventriculostomy and the monitoring of vasospasms by transcranial Doppler and CT or MR perfusion studies whenever necessary, as well as the treatment of the latter by medical and, when necessary, endovascular means. Patients living outside of Martinique needing elective services are managed through teleconsultation and travel to Martinique for their angiographies, elective treatments, and sometimes consultations. All the costs of travel for French citizens are covered by the French social security system.

### 2.2. Study Population—Inclusion and Exclusion Criteria

In this single-center, retrospective, descriptive study, all adult patients who were treated at the University Hospital of Martinique (CHUM) for intracranial aneurysms, ruptured or elective, from 1 January 2021 to 31 March 2024 were included. Data were retrospectively analyzed from a prospectively maintained database. The study protocol was approved by the institution’s ethical committee.

### 2.3. Data Collection and Analyses

All patients were managed according to international and national standards and established protocols. We retrospectively analyzed the demographics, clinical presentations, imaging and clinical data, postoperative examinations, and clinical follow-ups of the patients treated for intracranial aneurysm at the University Hospital of Martinique as described above.

### 2.4. Embolization Techniques and Patient Management

All cases were discussed between interventional neuroradiologists (INRists), neurosurgeons, anesthetists, and, for ruptured cases, ICU specialists. Decision for treatments was made with consensus.

The procedures were initially performed in the monoplane angiosuite, equipped with 3DRA (Allura, Philips Healthcare, Best, The Netherlands). Since July 2023, procedures have been performed in the new, state-of-the-art biplane angiosuite (Artis Icono, Siemens, Erlangen, Germany), which replaced the monoplane angiosuite. Therapeutic decisions were made with consensus between the INRist, neurosurgeon, and ICU doctor (for ruptured cases).

All patients were given an IV bolus of heparin (60 IU/kg) at the beginning of the procedure, after groin puncture, followed by a bolus of 1000 IU after 75 min, which was repeated after 60 min in cases of long interventions. All patients who underwent embolization techniques requiring a dual anti-platelet regimen were premedicated with 2 × 90 mg ticagrelor the evening before and the morning of the procedure. During the procedure, 250 mg of aspirin was given intravenously at the time of stent deployment. Patients were given 90 mg ticagrelor in the morning and evening and 75 mg aspirin at noon for 6 months post-procedure, followed by 6 months of 75 mg aspirin in the morning and evening. Patients susceptible to microbleeds were screened via MRI before any intervention, and the therapeutic strategy was adapted accordingly.

Digital subtraction angiography controls were scheduled at 3 to 6 months post-intervention, depending on the type of intervention, at 9 to 12 months, and at 18 months, followed by 24-month and 5-year controls. Clinical controls followed the same pattern.

### 2.5. Outcomes

The primary outcome was functional independence, defined as a modified Rankin scale score of 0 to 2 (mRS ≤ 2) at 4- to 6-month controls. The primary safety outcome was procedure-related morbidity. Secondary outcomes included angiographic outcomes at the end of the procedure and at the last angiographic outcome, as well as good clinical outcomes at 4 to 6 months for the acute rupture subgroup, defined as mRS ≤ 3.

### 2.6. Statistical Analysis

Qualitative data were described by population size and frequencies. Quantitative data were described by means, standard deviations, and percentiles after verification of the normality of the distribution with the D’Agostino–Pearson test; medians were reported in cases of non-normality of the distribution. We divided the cohort into two subgroups, one concerning patients with acutely ruptured aneurysms and one with patients treated electively for unruptured or recanalized intracranial aneurysms. Group comparisons were carried out using the Chi2 test or the Fisher exact test for the comparison of qualitative variables and the *t*-test for the comparison of quantitative variables. The McNemar test was used for paired data in order to evaluate clinical evolution. We proceeded to divide ruptured aneurysm cases into two groups, one which included air transfer and one which concerned patients from Martinique, in order to compare the clinical outcomes in the two cases. SPSS software (version 29) was used for the statistical analysis. The level of statistical significance was *p* ≤ 0.05.

## 3. Results

From 1 January 2021 to 31 March 2024, one hundred patients harboring 125 intracranial aneurysms were treated and included in the study. One patient was excluded from the study as they had to be transferred out due to social reasons. Overall, women constituted 81% of the population, while 19% were men (z-test = 5.2694, *p* < 0.00001); women constituted 79% of patients in the ruptured aneurysm group and 81% in the unruptured aneurysm group.

Regarding location, 60% of the cases were from Martinique and 40% involved air transfer. Furthermore, 97.4% of the patients had at least one risk factor and almost half (47.2%) had more than one risk factor. Hypertension, known or discovered, was present in 71% of the patients (Table 1).

Subgroup analysis based on ruptured versus unruptured aneurysms showed that ruptured aneurysms were significantly smaller in terms of maximal diameter (in mm) and had narrower necks than the non-ruptured ones. In total, 50% of the ruptured aneurysms were smaller than or equal to 5 mm and 80% were smaller than or equal to 7 mm, with a mean maximum diameter of 5.2 ± 2.1, which was significantly smaller than that of the elective cases (*p* = 0.004). Only 15 of the ruptured aneurysms were larger than 7 mm (19.7%), as shown in Table 2.

Sixty-eight percent (68%) of the cases of the cohort were mRS 0 to 2 at 4 to 6 month control. Subgroup analysis revealed autonomy at 4 to 6 months (mRS 0–2) of 60.4% of the acutely ruptured cases and of 89.7% of the elective cases. For the latter there was 0% of procedure related morbidity or mortality, while for the ruptured cases there was 9.2% procedure related complications (5.6% for the cohort); mortality at discharge was 10.4% for the cohort (Table 2 and Table 3).

Overall, 61.8% of patients had a modified WFNS score of III or higher at the initial evaluation (Table 1).

We divided the ruptured cases into two groups: one with good clinical outcomes, defined as an mRS score of 0 to 3 before tt, and one with bad clinical outcomes, defined as an mRS score of 4–6 at discharge and at 4–6 months. We compared the groups of mRS before with the groups of mRS at discharge and at 4–6 months. The comparison did not show a statistically significant difference among the groups (McNemar test *p* = 0.08 and *p* = 0.56, respectively).

The overall procedure-related complication rate was 5.6% (7/125). All the complications occurred in acutely ruptured cases (9.2%, 7/76), with three cases of periprocedural rupture in the monoplane angiosuite, two in the biplane angiosuite, and two procedure-related ischemic complications in the monoplane angiosuite. There was no statistically significant difference regarding the percentages of procedure-related complications in the monoplane and biplane angiosuites (*p* = 0.7). The median maximal diameter of the ruptured aneurysms where complications were observed was 5 mm [range 3 to 7], versus 5 mm [range 2 to 10] for the ones that did not have complications during the procedure; the difference was not statistically significant (Mann–Whitney test, *p* = 0.75)

Patients transferred by air were statistically comparable to those already in Martinique in terms of initial GCS and modified WFNS scores, as well as aneurysm and neck size, but they were significantly younger (57.9 ± 10.1 years for air transfer versus 58.9 ± 15.4 years in Martinique, *p* = 0.016). Four cases were not transferred within the selected period. They were similar in terms of mRS score at 4 to 6 months (Chi squared test for mRS values did not reveal a statistically significant difference, *p* = 0.5).

## 4. Discussion

**Figure 1:**
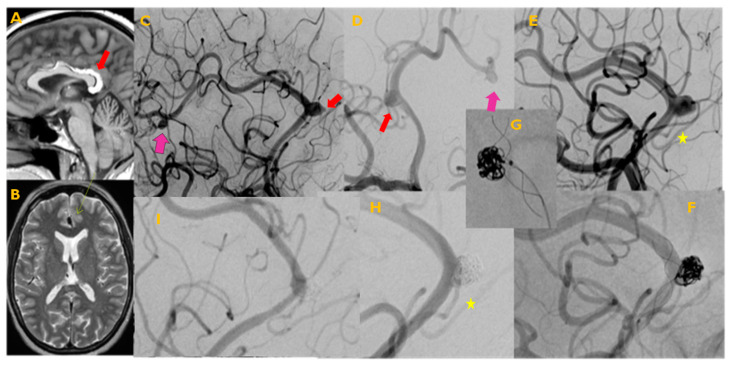
Patient in their late sixties, initial MRI performed for atypical headaches, absence of known cardiovascular risk factors. (**A**) FLAIR, sagittal plane, incidental finding of corpus callosum lipoma (red thick arrow); (**B**) axial T2 suspicion of IA over a signal void adjacent to the lipoma, (thin arrow); (**C**) digital subtraction angiography, lateral plane, showing the suspected pericallosal A3 aneurysm (small, thick arrow), with a prominent phlyctena at the dome, as well as a second, A4 aneurysm (big arrow); (**D**) oblique plane showing the same aneurysms, both with cortical branches coming out of the sacs; (**E**) working projection, asterisk, branch coming out of the aneurysm’s sac; (**F**,**G**) working projection, unsubtracted image showing stent-assisted coiling, with a stent LEO Baby 3 × 18 mm (Balt extrusion, Montmorency, France) and coils (Stryker, Kalamazoo, MI, USA); (**H**) angiographic result at the end of the procedure showing good wall apposition of the stent, occlusion of the aneurysm, and excellent permeability of the jailed branch (yellow asterisk); (**I**) DSA control at 6 months, showing occlusion of the aneurysm and complete patency of the jailed branch.

To the best of our knowledge, this is the first cohort of patients with intracranial aneurysms treated by endovascular means in the insular Caribbean region. The population was distinct in terms of risk factors. In accordance with previous studies on cardiovascular risk factors in the Antilles population, a higher prevalence of systemic hypertension (71%) was documented compared to other populations, such as in metropolitan France, where an incidence of 27% has been reported [2,8,9]. There was also an overrepresentation of patients with multiple intracranial aneurysms (35%) compared to other populations [8,10,11] (Figure 1).

The high incidence of multiple aneurysms may be partially attributed to the high prevalence of hypertension, as well as multiple risk factors—97% of the patients had at least one risk factor and around half of them had at least two risk factors. Systemic hypertension is associated with aneurysm growth, which has proven to be a very significant predictor of rupture [3,4]. Several mutations have been proven to be associated with familial forms and multiple aneurysms [12]. The insularity of the region may also play a role in the overexpression of genetic predisposing factors. The role and interaction of environmental risk factors and genetic predispositions have not been completely elucidated, but it seems that there is a genetic burden in the region, resulting in a higher incidence of multiple aneurysms. This is likely compounded by hemodynamic disturbances due to uncontrolled hypertension, which probably contributes to the higher incidence of multiple aneurysms and smaller size of ruptured aneurysms in the described population.

A recent study on the characteristics of very small ruptured IAs in a Korean population showed a very strong female predominance and a lower aspect ratio of IAs [13]. Even though there is no clear consensus on the sizing of intracranial aneurysms, in the study presented herein, the group of ruptured aneurysms had a significantly smaller mean maximal diameter than that of the elective group and included small aneurysms overall, in terms of both the ISUIA 2 (≤7 mm, 80% of the ruptured cases) and the UCAS classifications (≤5 mm, 50% of the ruptured cases) [14,15].

The present study reports an impressive female predominance (statistically significant, *p* ≤ 0.0001) in the ruptured and unruptured groups of aneurysms (81% in the cohort, 79% in the ruptured group, and 81% in the unruptured group). There is published evidence showing that women have a higher incidence of aneurysm rupture than men [16]. However, this predominance was not significant and, unlike in the present study, was reported only in ruptured aneurysms. The reasons for this female predominance have not yet been explained and warrant further investigation, particularly with genetic studies in order to search for X-linked mutations in our specific population. Further investigation will probably allow us to better understand the pathogenesis of IAs.

The dome-to-neck ratio was significantly smaller in the ruptured group in the present study. This is probably related to the fact that a part of the sac is actually a faux sac after IA rupture. This further accentuates the fact that in the Antilles population, small and very small aneurysms do rupture, likely at a higher rate than in other continental populations [8,14,17].

Clinical outcomes for both acutely ruptured and elective cases were very satisfactory [17,18]. In the elective cases, mRS scores were excellent at 4 to 6 months, without modification, as compared to the pre-treatment mRS scores, and there were no procedure-related complications. The clinical outcomes presented herein seem more favorable than those reported in the ARETA study (1.8% periprocedural aneurysm ruptures; 10.4% iatrogenic emboli) and in a recent meta-analysis (2.6% periprocedural ruptures, 7.6% iatrogenic emboli, and 1.2% procedure-related deaths) [8,19,20]. This is probably related to the fact that in the given period, almost all elective cases were performed by a single, experienced operator (C.I.), with no learning curve involved due to the absence of fellows or practitioners in training.

In a recently published study, the authors reported a higher risk of procedure-related aneurysm rupture during embolization for aneurysms with a maximal diameter of less than 5 mm [8]. This may partially explain why the procedure-related rupture rate in our group of acutely ruptured aneurysms was in the higher range. Nevertheless, we did not find a statistically significant correlation between size and procedure-related rupture in our group of ruptured cases. In the acutely ruptured group, procedure-related complications included 6.6% periprocedural aneurysm ruptures and 2.6% thromboembolic complications. Procedure-related rupture rates seem to vary in the literature, ranging from 3.1% to 7.5%, but the results presented herein (6.6% in the ruptured subgroup, 0.2% for the cohort) are acceptable [8,14,17,20,21].

Procedure-related ischemic events in the present study seem to be less common than in other studies and only occurred in ruptured cases (1.6% in the cohort, 2.6% in the acutely ruptured group), as opposed to 10 to 13% in published data [8,14,19,20,21]. More ischemic complications were documented in the monoplane angiosuite (two cases versus zero in the biplane angiosuite); however, this did not reach statistical significance. The mortality rate was high in the acutely ruptured group compared to other studies [19]. The group of ruptured cases presented herein had very serious clinical presentations initially: 49% of the acutely ruptured cases had a GCS score of thirteen or lower on the initial evaluation at admission, as compared to 6% in the ISAT study [17]. Only 38.2% of the patients had an mWFNS classification of I or II (Table 1). Even though the patients with SAH in the cohort were in more severe initial clinical condition than in other studies, more than half of them were autonomous at 4 to 6 months post-intervention.

Angiographic outcomes included very satisfactory occlusions (Roy Raymond I and II) in 91% of cases post-procedure and 94% of cases at the last angiographic control, in cases which included coiling, remodeling, or stent-assisted coiling. These percentages are higher compared to published data [8]. Regarding the angiographic evolution of the cases with flow diverters, these seemed very satisfactory. At the last angiographic follow-up of more than a year (mean delay = 13.2 ± 7.8 months), all cases showed regional remodeling grades of B to D on the OKM scale; thus, the aneurysm walls were not opacified and were exhibiting favorable evolution towards stable remodeling [21]. There were no re-ruptures during the follow-up period for all aneurysms in the cohort.

Forty percent of the cases were transfers to Martinique from the other Antilles regions, while sixty percent were treated without need for air transfer. Air transfer was a safe and effective measure, allowing us to avoid transatlantic air transfers to mainland France, which was the standard of care until 2021 [22]. Even though it was considered an effective practice at the time due to the absence of better standards, longer air transfers in cases of SAH are associated with worse patient prognosis, especially in terms of mortality [23]. The development of a complete INR center onsite represents a game changer for patient care. All patients are now routinely followed and treated electively whenever necessary, which was not the case before 2021.

The patients from Martinique seemed to be younger compared to those transferred by air from other regions. Even though there was no statistically significant difference in primary outcomes between patients transferred by air and those who were already in Martinique, there was a tendency for better clinical outcomes for the transferred patients. The initial GCS and WFNS scores of the transferred patients were statistically comparable, thus indicating that the patients transferred by air were not in a better clinical state than those transferred by road. There were only four patients not transferred during the studied period. This finding needs more research in order to find out whether older patients are not transferred to the tertiary hospitals at all in the other areas, except for Martinique, or whether there is another reason for this age difference. A study is underway on this subject.

The use of air transfers for IA treatments in remote areas, insular or not, whether it is the patient or the practitioner that is transferred, remains underreported in the literature and is dependent on factors including regional resources and security system particularities [24,25]. To the best of our knowledge, this is the first worldwide model of patients transferred for elective and ruptured intracranial aneurysms, with the fees covered by public insurance. The system allows for optimal treatment and follow-up of patients at a high-level expert center, just as in mainland France, even for patients living in an insular setting. It simultaneously ensures the maintenance of expertise in the high-volume center of the University Hospital of Martinique.

Nevertheless, this example is based on a public system of care, with fees covered for air transfers for all patients. That being said, private security systems may also use this type of system, given the quality of care and the improvement in clinical outcomes for the patients. The positive experience presented here, both in terms of clinical outcomes and healthcare organization, is largely based on the almost exclusive use of neuroendovascular techniques. Although there is no general consensus about this, it should be acknowledged that the field of neurosurgery is moving in this direction. Due to recent technological advances, “traditional” aneurysm embolization has been on the rise in the last ten years, as opposed to microsurgery [26].

In the real-life study presented herein, the arrival of a biplane angiosuite in June 2023 allowed for a switch in treatments from the monoplane to the new biplane system. The comparison of the complication rates for IA treatment did not show statistically significant differences between the treatments performed in the monoplane versus the biplane angiosuite. There were two procedure-related ruptures in each of the angiosuites, all in cases of acutely ruptured aneurysms. There were no procedure-related ischemic complications in the biplane angiosuite, compared to two ischemic complications in the monoplane, but this difference was not statistically significant either.

Embolizations for IAs were safe and effective with the monoplane angiosuite in the cohort presented here; the French Antilles population had been covered for thrombectomy since 2020 [7] and for intracranial aneurysm treatments since 2021. Nevertheless, the arrival of the biplane angiosuite represented a significant landmark since it allowed for an increase in and diversification of the therapeutic arsenal of the INR center. The latter became, in 2023, a complete tertiary INR center, with treatments such as curative elective and ruptured AVM treatments in adults and children, and has since attracted international interest.

The first tertiary INR center in the Antilles has a vocation to promote health for patients suffering from vascular pathologies of the CNS, as well as to provide scientific and research data on neurovascular pathologies and promote teaching and training in INR. For these three goals, the databases of the INR cohorts are updated regularly with the clinical and angiographic data of the patients in order to be able to provide longer-term data and evaluate the mid- and long-term outcomes of the regional strategy.

The tertiary INR center in the French Antilles allows for the proper continuous education of healthcare providers, especially family doctors. Nowadays, practitioners routinely address unruptured cases for evaluation and FU to the center. Before 2021, patients with unruptured aneurysms were not systematically followed by an expert interventional neuroradiologist. They were sometimes sent to mainland France or visited a specialist there on their own initiative, but this was not consistent, resulting in more ruptured cases. Presently, international collaborations are underway in order to improve awareness and know-how in the greater region and provide state-of-the-art care for Caribbean patients.

By national decree, there are a limited number of complete INR centers per million people in order to maintain a high level of expertise and technical ability. The team in Martinique is considering several options of future interaction with other sites in the French Antilles in order to maintain equity of care, including transfer of patients as well as treating teams whenever necessary and exploiting the opportunities telemedicine has to offer. Our priority is to provide equity of access to treatment for all Antilles–French Guyana citizens and achieve fluid collaboration with neighboring nations in order to elevate the standards of care in the insular Caribbean.

In light of recent data highlighting the scarcity of INR-thrombectomy resources in the Caribbean [5], the presence of the complete INR center at the CHUM seems to be of great importance for the development of access to INR treatments. At this stage, reinforcing international collaborations between the island nations of the Caribbean is paramount, including significant work on the health insurance interface, in order to be able to seamlessly transfer patients from one center to the other.

At the same time, scientific collaborations are equally important in order to promote training exchanges and increase the number of qualified INR practitioners in the area, as well as promote research and an overall better understanding of the particularities of our populations, with a vision to provide tailored prevention and treatment strategies to our patients regarding neurovascular pathologies. A synergy between hospitals, universities, governments, and implicated parties in general is necessary at this stage in order to address the important inequalities in access to INR care in the insular Caribbean.

## 5. Conclusions

Patients with ruptured intracranial aneurysms in the French Antilles presented with poorer clinical features compared to other cohorts. Patients had an overexpression of predisposing risk factors, especially systemic hypertension, and the majority (80%) were women. The initiation of a tertiary INR center in Martinique with air transfer facilities was safe and effective. Elective treatments are safe and effective and will contribute to alleviating the burden of IA-related disability in the region.

## Figures and Tables

**Table 1:** Patient demographics.

	Cohort	
Number of patients	100	
Number of aneurysms	125	
Mean diameter (in mm, male/female)	5.53/5.89	*p* = 0.63
Age (arithmetic mean ± SD)	56.7 ± 12.2	
Sex (%, m/f)	19/81	*p* < 0.00001
Mean number of aneurysms per patient	1.8 ± 1.1	
Origin		
Martinique	60 (60%)	
Guadeloupe	21 (21%)	
French Guyana	13 (13%)	
Saint Martin	2 (2%)	
French Mainland	1 (1%)	
Abroad	3 (3%)	
Known risk factors		
Hypertension	71%	
Obesity	22%	
Diabetes	11%	
Smoking	32%	
Dyslipidemia/hypercholesterolemia	33%	
Family history	11%	
Multiple aneurysms	35%	
Cumulative number of known risk factors		
Zero	2 (2.6%)	
One	38 (49.2%)	
Two	18 (23.4%)	
Three	12 (15.6%)	
Four	5 (6.5%)	
Five	2 (2.6%)	

**Table 2:** Aneurysm characteristics and data.

	Cohort	Acutely Ruptured	Elective	*p*-Value
Number of aneurysms	125	76 (60.8%)	49 (39.2%)	
Maximum diameter	5.8 ± 3.6	5.2 ± 2.1	6.8 ± 4.9	0.0390
Neck diameter	3.5 ± 1.8	3.0 ± 1.2	4.1 ± 2.4	0.0045
Dome-to-neck ratio	1.4 ± 0.4	1.4 ± 0.4	1.3 ± 0.3	0.2924
Aspect ratio	1.7 ± 0.6	1.8 ± 0.6	1.6 ± 0.6	0.1012
Location of aneurysm				
MCA bifurcation	22 (17.6%)	15 (19.7%)	7 (14.3%)	0.45
Acom	20 (16.0%)	18 (23.7%)	2 (4.1%)	0.003
A1–A2	7 (5.6%)	5 (6.6%)	2 (4.1%)	0.60
Carotido ophthalmic	23 (18.4%)	3 (3.9%)	20 (40.8%)	<0.0001
Pcom	27 (21.6%)	21 (27.6%)	6 (12.2%)	0.04
ACha	4 (3.2%)	1 (1.3%)	3 (6.1%)	0.19
PICA	1 (0.8%)	1 (1.3%)	0 (0%)	0.61
Basilar top	2 (1.6%)	1 (1.3%)	1 (2.0%)	0.78
Carotid T	9 (7.2%)	6 (7.9%)	3 (6.1%)	0.74
Superior cerebellar	1 (0.8%)	1 (1.3%)	0 (0%)	0.61
Pericallosal	6 (4.8%)	4 (5.3%)	2 (4.1%)	0.80
ICA cavernous	3 (2.4%)	0 (0%)	3 (6.1%)	0.06
Type of aneurysm				
Saccular	123 (98.4%)			
Blister	2 (1.6%)	2	0	
Manifestation				
Fortuitous	34 (27.20%)			
SAH	76 (60.80%)			
Nerve compression	2 (1.60%)			
Wall inflammation/instability	1 (0.80%)			
Fischer				
1		1 (1.31%)	Not concerned	
2		1 (1.31%)		
3		16 (21.05%)		
4		58 (76.31%)		
First treatment/recanalization	113 (90.40%)	Not concerned	37 (75.5%)	
	12 (9.60%)		12 (24.5%)	
Type of intervention				
Simple colling	78 (62.4%)	62 (81.6%)	16 (32.7%)	<0.0001
Balloon remodeling	16 (12.8%)	10 (13.2%)	6 (12.2%)	0.90
Stent + coils	4 (3.2%)	0 (0%)	2 (4.1%)	0.15
FD + coils	7 (5.6%)	0 (0%)	7 (14.3%)	0.001
FD no coils	18 (14.4%)	2 (2.6%)	18 (36.7%)	<0.0001
Surgery	1 (0.8%)	1 (1.3%)	0 (0%)	0.61
Abstention	1 (0.8%)	1 (1.3%)	0 (0%)	0.61
Number of cases in monoplane and biplane angiosuite	Monoplane	Monoplane	Monoplane	
	85 (68%)	53 (69.7%)	32 (65.3%)	0.61
	Biplane	Biplane	Biplane	
	40 (32%)	23 (30.3%)	17 (34.7%)	0.61

**Table 3:** Clinical status and outcomes.

	Cohort	Acutely Ruptured	Elective	*p*
mRS before tt				
0	37 (29.83%)	0	37 (75.51%)	<0.0001
1	32 (25.80%)	26 (34.66%)	6 (12.24%)	<0.0001
2	12 (9.68%)	11 (14.67%)	1 (2.04%)	0.02
3	19 (15.32%)	14 (18.67%)	5 (10.20%)	0.22
4	11 (8.87%)	11 (14.67%)		
5	13 (10.48%)	13 (17.33%)		
	One missing variable	One missing variable		
mRS discharge				
0	47 (37.6%)	10 (13.2%)	37 (75.51%)	<0.0001
1	29 (23.2%)	23 (30.3%)	6 (12.24%)	0.02
2	7 (5.6%)	6 (7.9%)	1 (2.04%)	0.19
3	11 (8.8%)	6 (7.9%)	5 (10.20%)	0.66
4	17 (13.6%)	17 (22.4%)		
5	1 (0.8%)	1 (1.3%)		
6	13 (10.4%)	13 (17.1%)		
mRS 4–6 months				
0	52 (41.60%)	15 (19.7%)	37 (75.51%)	<0.0001
1	25 (20.00%)	19 (25.0%)	6 (12.24%)	0.08
2	8 (6.40%)	7 (9.2%)	1 (2.04%)	0.12
3	14 (11.20%)	9 (11.8%)	5 (10.20%)	0.80
4	11 (8.80%)	11 (14.5%)		
5	1 (0.80%)	1 (1.3%)		
6	14 (11.20%)	14 (18.4%)		
Initial GCS		Mean GCS: 11.6 (median: 13: min: 4: max: 15)	GCS: 15 for all	
4	4 (3.39%)	4 (5.3%)		
5	3 (2.54%)	3 (3.9%)		
6	1 (0.85%)	1 (1.3%)		
7	6 (5.08%)	6 (7.9%)		
8	3 (2.54%)	3 (3.9%)		
9	2 (1.69%)	2 (2.6%)		
10	4 (3.39%)	4 (5.3%)		
11	3 (2.54%)	3 (3.9%)		
12	4 (3.39%)	4 (5.3%)		
13	10 (8.47%)	10 (13.2%)		
14	7 (5.93%)	5 (6.6%)		
15	71 (60.17%)	24 (31.6%)		
	(7 missing data: sedated pt, not evaluable)	(7 missing data: sedated pt, not evaluable)		
mWFNS				
1	24 (31.6%)			
2	5 (6.6%)			
3	10 (13.2%)			
4	22 (28.9%)			
5	8 (10.5%)			
	(7 missing data: sedated pt, not evaluable)			
External ventriculostomy		43.4% (33/76)	0	
Before embo		38.2% (29/76)		
After embo		5.2% (4/76)		
No ventriculostomy		56.6% (43/76)		
Procedure-related complications	5.6% (7/125)	9.2% (7/76)	0% (0/49)	
Aneurysm rupture	0.2% (5/125)	6.6% (5/76)	0	
Iatrogenic arterial occlusion	1.6% (2/125)	2.6% (2/76)	0	
Complications related to the SAH	20% (25/125)	32.8% (25/76)	0% (0/49)	
Hydrocephalus	5.6% (7/125)	9.2% (7/76)	0	
Vasospasm	14.4% (18/125)	23.6% (18/76)	0	
Procedure-related morbidity rate	5.6% (7/125)	9.2% (7/76)	0%	
Mortality rate	10.4%	10.4%	0%	
Angiographic outcomes at end of the procedure				
Roy Raymond I	66 (53.2%)	52 (69.3%)	14 (28.6%)	<0.0001
Roy Raymond II	24 (19.4%)	16 (21.3%)	8 (16.3%)	0.53
Roy Raymond IIIa	11 (8.9%)	6 (8%)	5 (10.2%)	0.66
Roy Raymond IIIb	6 (4.8)%	1 (1.3%)	5 (10.2%)	0.04
OKM A	8 (6.5%)		8 (16.3%)	
OKM B	9 (7.3%)		9 (18.4%)	
Angiographic outcomes at last angiographic control				
Roy Raymond I	12 (26.7%)	28 (58.3%)	12 (26.7%)	0.15
Roy Raymond II	10 (22.2%)	17 (35.4%)	10 (22.2%)	0.81
Roy Raymond IIIa	4 (8.9%)	3 (6.3%)	4 (8.8%)	0.35
Roy Raymond IIIb	3 (6.7%)	0 (0%)	3 (6.7%)	0.06
OKM A	0 (0%)		0 (0%)	
OKM B	1 (2.2%)		1 (2.2%)	
OKM C	6 (13.3%)		6 (13.3%)	
OKM D	9 (20.0%)		9 (20.0%)	
		Missing 28	Missing 4	
Delay in procedure to last angiographic control in months (arithmetic mean ± SD)	11.3 ± 7.4	9.6 ± 6.7	13.2 ± 7.8	0.007

## Data Availability

The original contributions presented in this study are included in the article. Further inquiries can be directed to the corresponding author.

## Abbreviations

The following abbreviations are used in this manuscript:

IA	Intracranial aneurysm
SAH	Subarachnoid hemorrhage
DSA	Digital subtraction angiography
INR	Interventional neuroradiology
HEMS	Helicopter emergency medical service
mRS	Modified Rankin scale
mWFNS	Modified World Federations Neurosurgical Societies score
